# High-Quality Genome Assembly and Annotation for *Plasmodium coatneyi*, Generated Using Single-Molecule Real-Time PacBio Technology

**DOI:** 10.1128/genomeA.00883-16

**Published:** 2016-09-01

**Authors:** Jung-Ting Chien, Suman B. Pakala, Juliana A. Geraldo, Stacey A. Lapp, Jay C. Humphrey, John W. Barnwell, Jessica C. Kissinger, Mary R. Galinski

**Affiliations:** a International Center for Malaria Research, Education and Development, Emory Vaccine Center, Yerkes National Primate Research Center, Emory University, Atlanta, Georgia, USA; b Department of Genetics, Institute of Bioinformatics, Center for Tropical and Emerging Global Diseases, University of Georgia, Athens, Georgia, USA; c Biosystems Informatics & Genomics, René Rachou Research Center (CPqRR-FIOCRUZ), Belo Horizonte, Minas Gerais, Brazil; d Malaria Branch, Division of Parasitic Diseases and Malaria, Centers for Disease Control and Prevention, Atlanta, Georgia, USA; e Division of Infectious Diseases, Department of Medicine, Emory University, Atlanta, Georgia, USA; f Malaria Host–Pathogen Interaction Center, Emory University, Atlanta, Georgia, USA

## Abstract

*Plasmodium coatneyi* is a protozoan parasite species that causes simian malaria and is an excellent model for studying disease caused by the human malaria parasite, *P. falciparum*. Here we report the complete (nontelomeric) genome sequence of *P. coatneyi* Hackeri generated by the application of only Pacific Biosciences RS II (PacBio RS II) single-molecule real-time (SMRT) high-resolution sequence technology and assembly using the Hierarchical Genome Assembly Process (HGAP). This is the first *Plasmodium* genome sequence reported to use only PacBio technology. This approach has proven to be superior to short-read only approaches for this species.

## GENOME ANNOUNCEMENT

A *Plasmodium* genome sequence was published initially in 2002, for *P. falciparum* (1). Genome sequences for several other primate *Plasmodium* species have followed (1–6), but none has yet been generated using only PacBio technology (6). *Plasmodium coatneyi*, which infects *Macaca mulatta* (rhesus macaques) and serves as a model of *P. falciparum* (7, 8), was chosen as a test platform for PacBio sequencing. A preliminary draft of the *P. coatneyi* genome based on short-read (<500 bp) sequence technology is available in the NCBI database (PRJNA233970). Although the overall “big picture” can be gained from this genome assembly, there are over 500 sequence gaps distributed throughout the parasite’s estimated 14 nuclear chromosomes. Like *P. falciparum*, the *P. coatneyi* genome has numerous repetitive sequences and complex multi-gene families which present major difficulties that have prohibited nontelomeric genome assembly with closure using only short-read technologies. Gaps prevent reliable gene content analysis, genetics and reference-based gene expression analyses, all of which are critical for understanding *Plasmodium* and disease progression. We have implemented PacBio (RSSMRT) sequence technology to tackle these issues.

Genomic DNA was extracted from *ex vivo* matured schizont-stage parasites with a Qiagen DNA blood midi kit. The gDNA was further purified with a PowerClean DNA cleanup kit (Mo Bio Laboratories). Five micrograms of gDNA were subsequently used for library preparation. SMRTbell DNA libraries (Pacific Biosciences) were constructed according to the PacBio standard protocol with the BluePippin size-selection system (Sage Science). Sequence was generated on a PacBio RSII instrument using P6-C4 chemistry. Following cleaning, the mean assembled subread length is 5,824 bp; the *N*_50_ is 7,257; the total number of bases is 1,792,197,364 and the total number of reads is 257,557. HGAP3 (9) *de novo* assembly was performed using the Amazon EC2 cloud SMRT portal. The error correction module was defined as minimum subread length of 100 bp, a minimum read quality of 0.80, and a minimum read length of 6,000 bp. Following host (*M. mulatta*) contig removal, 15 nuclear contigs, one mitochondrial contig, and one apicoplast contig remained (51.42× average coverage). Contig identity and synteny were evaluated via BLASTn and progressive MAUVE algorithms (10) using the *P. knowlesi* genome from GeneDB (3) as the reference. Two suspected interchromosomal rearrangements occurring within gene family sequences located on Chr4/Chr13 and Chr12/Chr14 could not be validated by PCR, suggesting these sequences may in fact be correct as presented here.

*De novo* gene prediction was performed using SNAP (11) and Augustus (12) for gene calls in the MAKER2 (13) genome annotation tool. The *P. vivax* and *P. knowlesi* predicted proteomes were included as evidence. In total, 5,516 protein-encoding genes were predicted, including up to 112 *SICAvar* genes. The complete annotated mitochondrial and apicoplast genomes are also included in this report. The annotation was validated with *P. coatneyi* RNA-Seq data, Uniprot, KEGG and OrthoMCL Orthology, and InterProScan5 (14–18). 5,060 genes have strong evidence of synteny.

### Accession number(s).

The fourteen chromosome sequences were deposited at NCBI (BioProject PRJNA315987) under accession numbers CP016239 to CP016252 and provided to PlasmoDB (19).
